# Supplementary material for: Neural Networks as Entropic Systems: Applications in Digital Pathology
Source: bioRxiv. 2026 Feb 2:2026.01.30.702864. Preprint. [Version 1] doi: 10.64898/2026.01.30.702864 (PMC12889516; doi:10.64898/2026.01.30.702864)
Supplement: Supplement 1 [file NIHPP2026.01.30.702864v1-supplement-1.pdf]

bioRxiv preprint doi: https://doi.org/10.64898/2026.01.30.702864; this version posted February 2, 2026. The copyright holder for this preprint (which was not certified by peer review) is the author/funder, who has granted bioRxiv a license to display the preprint in perpetuity. It is made available under aCC-BY 4.0 International license.

Supplementary: Proofs and Formal Statements

12 Use Of Generative Artificial Intelligence

The author used ChatGPT for grammar, punctuation, and formatting.

=

Setup

Let $L(\theta)$ be the empirical risk for parameters $\theta \in \mathbb{R}^d$, and let $g(\theta) = \nabla_\theta L(\theta)$. Per-batch gradients are $g_b(\theta)$ with $\mathbb{E}[g_b(\theta)] = g(\theta)$ and $\Sigma(\theta) = \mathrm{Cov}[g_b(\theta)]$. Activations at layer ℓ for input x are $a_\ell(x) \in \mathbb{R}^{d_\ell}$ with forward map $a_\ell = \phi_\ell(W_\ell a_{\ell-1})$, where W_ℓ are layer weights and ϕ_ℓ is (piecewise) smooth.

bioRxiv preprint doi: https://doi.org/10.64898/2026.01.30.702864; this version posted February 2, 2026. The copyright holder for this preprint (which was not certified by peer review) is the author/funder, who has granted bioRxiv a license to display the preprint in perpetuity. It is made available under aCC-BY 4.0 International license.

Non-degenerate model. We assume (i) the network contains at least two coupled parameters and two coupled units per layer, (ii) the loss and architecture are not separable across coordinates, so the Hessian $H(\theta) = \nabla^2_\theta L(\theta)$ is not diagonal and some backprop Jacobians have nonzero off-diagonal entries.

Proposition 1 (Weights are not mutually independent). *Unless the model and loss are fully separable across coordinates, weight updates are statistically dependent within an epoch.*

Consider one SGD step (fixed learning rate η and batch size S):

$$\theta^+ = \theta - \eta\, g_b(\theta).$$

Let $\Delta\theta = \theta^+ - \theta = -\eta\, g_b(\theta)$. The covariance of updates is

$$\mathrm{Cov}[\Delta\theta] = \eta^2\, \mathrm{Cov}[g_b(\theta)] = \eta^2\, \Sigma(\theta).$$

If weights were mutually independent in their updates, $\mathrm{Cov}[\Delta\theta]$ would be diagonal. Under the non-degeneracy assumption, backprop couples coordinates, so $\Sigma(\theta)$ has nonzero off-diagonals. Hence $\mathrm{Cov}[\Delta\theta]$ has nonzero off-diagonals and coordinates are dependent. Equivalently, at the level of expected drift, $H(\theta)$ having off-diagonals implies coupled deterministic motion, and at the noise level, off-diagonal Σ implies coupled stochastic motion. Therefore weights are not mutually independent.

Proposition 2 (Activations are not mutually independent and are rankable by correlation/connectivity). *For a fixed distribution of inputs, within a given layer ℓ, the activation vector a_ℓ has a non-diagonal covariance matrix generically, and units can be ranked by correlation strength and graph connectivity.*

Write $z_\ell = W_\ell a_{\ell-1}$ and $a_\ell = \phi_\ell(z_\ell)$. If ϕ_ℓ is linear in a neighborhood and $a_{\ell-1}$ has

bioRxiv preprint doi: https://doi.org/10.64898/2026.01.30.702864; this version posted February 2, 2026. The copyright holder for this preprint (which was not certified by peer review) is the author/funder, who has granted bioRxiv a license to display the preprint in perpetuity. It is made available under aCC-BY 4.0 International license.

covariance $\Sigma_{a_{\ell-1}}$, then

$$\mathrm{Cov}[a_\ell] \approx W_\ell \, \Sigma_{a_{\ell-1}} \, W_\ell^\top,$$

which is non-diagonal unless W_ℓ is a signed permutation and $\Sigma_{a_{\ell-1}}$ is diagonal in the same basis. With nonlinear ϕ_ℓ, the delta method yields $\mathrm{Cov}[a_\ell] \approx J_\phi \, \mathrm{Cov}[z_\ell] \, J_\phi^\top$ with J_ϕ diagonal only if all units are at identical slopes; generically $\mathrm{Cov}[a_\ell]$ stays non-diagonal. Hence activations are not mutually independent.

Define the empirical correlation matrix R_ℓ by normalizing $\mathrm{Cov}[a_\ell]$ to unit variances. Build an undirected weighted graph $G_\ell = (V_\ell, E_\ell)$ with vertices the units and edge weights $w_{ij} = |R_{\ell,ij}|$ (or thresholded). Standard centralities (degree/strength $s_i = \sum_j w_{ij}$, eigenvector centrality, betweenness) induce a total preorder on V_ℓ. Therefore units are rankable by correlation strength and by graph connectivity.

Theorem 3 (Boltzmann stationary law holds under constant diffusion, conservative drift, and small-step regime). *Consider the continuous-time limit of SGD in parameter space*

$$d\theta_t = -\nabla L(\theta_t) \, dt + \sqrt{2D} \, dB_t,$$

where D is a constant scalar (equivalently $\Sigma(\theta) \equiv 2SD\,I$) and B_t is standard Brownian motion. If the drift is conservative ($-\nabla L$), then any stationary density that satisfies detailed balance is

$$p_\infty(\theta) \;\propto\; \exp\Big(-\frac{L(\theta)}{D}\Big).$$

The Fokker–Planck equation is

$$\partial_t p = \nabla \cdot \big(p \, \nabla L\big) + D \, \Delta p.$$

At stationarity and under detailed balance (zero probability current), $0 = \nabla \cdot \big(p_\infty \nabla L + D \, \nabla p_\infty\big)$, which for smooth, positive p_∞ integrates to $D \, \nabla \log p_\infty = -\nabla L$. Hence $\log p_\infty = -L/D + \mathrm{const}$, i.e., $p_\infty \propto e^{-L/D}$.

bioRxiv preprint doi: https://doi.org/10.64898/2026.01.30.702864; this version posted February 2, 2026. The copyright holder for this preprint (which was not certified by peer review) is the author/funder, who has granted bioRxiv a license to display the preprint in perpetuity. It is made available under aCC-BY 4.0 International license.

Remarks. If diffusion is constant but anisotropic ($d\theta_t = -\nabla L\, dt + G\, dB_t$ with $GG^\top = 2A$ constant), then $p_\infty(\theta) \propto e^{-2\phi(\theta)}$ with $\nabla\phi = A^{-1}\nabla L$; if A depends on θ or if momentum/normalization layers induce non-conservative drift, detailed balance fails and the simple Boltzmann form need not hold. The diffusion approximation itself requires small learning rate, fixed batch size within the epoch, and i.i.d. sampling.

Theorem 4 (Entropy decreases as modularity/within-block correlation increases, under fixed marginals). *Let $X \in \mathbb{R}^d$ be zero-mean Gaussian with covariance C and fixed diagonal* $\operatorname{diag}(C) = \sigma^2$ *(fixed marginal variances). The differential entropy is*

$$h(X) = \tfrac{1}{2}\log\big((2\pi e)^d \det C\big).$$

Then $h(X)$ is maximized when coordinates are independent (C diagonal). In particular, for a fixed community partition, increasing within-community correlations (i.e., stronger modularity) strictly reduces $\det C$ *and thus reduces $h(X)$.*

By Hadamard's inequality, for any positive definite C,

$$\det C \;\le\; \prod_{i=1}^{d} C_{ii},$$

with equality iff C is diagonal (i.e., coordinates are uncorrelated). Fixing the diagonal entries $C_{ii} = \sigma_i^2$, the product on the right is constant, so $\det C$ is maximized at independence and strictly decreases as off-diagonal magnitudes grow. Stronger modularity means larger within-block correlations (larger $|C_{ij}|$ for i, j in the same community) while keeping the diagonal fixed; hence $\det C$ strictly decreases and $h(X)$ strictly decreases.

Corollary (Modularity vs. entropy, graph view). Let R be the correlation matrix of X (unit diagonal). The Newman–Girvan modularity Q for a fixed partition increases as within-block $|R_{ij}|$ increase and cross-block entries decrease. Since $h(X) = \frac{1}{2}\log\big((2\pi e)^d \det(\operatorname{Diag}(\sigma)\, R\, \operatorname{Diag}(\sigma))\big) = \text{const} + \frac{1}{2}\log\det R$, and $\det R$ decreases with larger within-block correlations, we obtain

bioRxiv preprint doi: https://doi.org/10.64898/2026.01.30.702864; this version posted February 2, 2026. The copyright holder for this preprint (which was not certified by peer review) is the author/funder, who has granted bioRxiv a license to display the preprint in perpetuity. It is made available under aCC-BY 4.0 International license.

$Q\uparrow\Rightarrow\ \det R\downarrow\Rightarrow\ h\downarrow.$

Optional local OU characterization (for diagnostics). Near a minimizer θ with Hessian $H\succ 0$ and constant diffusion (within an epoch),

$$d\Delta_t = -H\Delta_t\,dt + \sqrt{2D}\,dB_t, \qquad \Delta_t = \theta_t - \theta^{\cdot}$$

The stationary covariance C solves $HC + CH^\top = 2D\,I$, giving $\mathrm{Var}(\langle v_i, \Delta_t\rangle) = D/\lambda_i$ along Hessian eigenvector v_i with eigenvalue $\lambda_i > 0$. Thus flatter directions (smaller λ_i) exhibit larger equilibrium variance; adding correlation (modularity) lowers $\det C$ under fixed marginals, consistent with Theorem 4.

Summary of conditions for the Boltzmann claim.

- Fixed learning rate within the epoch (small step), fixed batch size; i.i.d. batches.
- Conservative drift $-\nabla L$ (no momentum/preconditioning in the claim, or incorporate them explicitly).
- Constant (locally) diffusion tensor within the epoch; isotropic for the simple $e^{-L/D}$ form.
- Local basin (linearization valid) for OU/Lyapunov diagnostics.

Standing assumptions (intra-epoch, late training). Small step size; fixed batch size; plain SGD; i.i.d. batches. For local results, linearize around a basin point θ with $H := \nabla^2 L(\theta^{)\succ 0}$. Gradient-noise covariance is locally constant: $\Sigma(\theta)\approx\Sigma_0$.

[Non-diagonal Hessian $\Rightarrow$ coupled deterministic drift] Let $L:\mathbb{R}^d\to\mathbb{R}$ be twice continuously differentiable. Consider the gradient flow $\dot\theta(t) = -\nabla L(\theta(t))$ and its linearization near θ with $H = \nabla^2 L(\theta^{)}$. If H has any off-diagonal entry $H_{ij}\neq 0$ for some $i\neq j$, then the i-th coordinate of the drift depends on (at least) the j-th coordinate; in particular, the deterministic dynamics are not coordinate-wise independent.

bioRxiv preprint doi: https://doi.org/10.64898/2026.01.30.702864; this version posted February 2, 2026. The copyright holder for this preprint (which was not certified by peer review) is the author/funder, who has granted bioRxiv a license to display the preprint in perpetuity. It is made available under aCC-BY 4.0 International license.

Linearizing at θ with $\Delta(t) = \theta(t) - \theta$ gives $\dot{\Delta}(t) = -H\,\Delta(t)$. Component-wise, $\dot{\Delta}_i(t) = -\sum_{k=1}^{d} H_{ik}\,\Delta_k(t)$. If $H_{ij} \neq 0$ for some $j \neq i$, then $\dot{\Delta}_i$ depends on Δ_j, hence the coordinates are coupled.

[OU stationary covariance $\Leftrightarrow$ Lyapunov equation] Consider the linear SDE (Ornstein–Uhlenbeck) in parameter space

$$d\Delta_t = -H\Delta_t\,dt + G\,dB_t,$$

with $H \in \mathbb{R}^{d\times d}$ Hurwitz (spec(H) in the open right half-plane) and diffusion $Q := GG^\top \succeq 0$ constant. If a stationary Gaussian law exists with covariance $C = \mathbb{E}[\Delta_t\Delta_t^\top]$, then

$$HC + CH^\top = Q.$$

Conversely, if H is Hurwitz and $Q \succeq 0$, there is a unique $C \succeq 0$ solving the Lyapunov equation, and the OU process is stationary Gaussian with covariance C.

Apply Itô to $\Delta_t\Delta_t^\top$:

$$d(\Delta\Delta^\top) = (d\Delta)\Delta^\top + \Delta(d\Delta)^\top + (d\Delta)(d\Delta)^\top = (-H\Delta\,dt + G\,dB)\Delta^\top + \Delta(-\Delta^\top H^\top\,dt + dB^\top G^\top) + G\,dB\,dB^\top G^\top [illegible]$$

Taking expectations and using $\mathbb{E}[dB] = 0$, $\mathbb{E}[dB\,dB^\top] = I\,dt$:

$$d\,\mathbb{E}[\Delta\Delta^\top] = \big(-H\,C - C\,H^\top + Q\big)\,dt.$$

At stationarity $d\,\mathbb{E}[\Delta\Delta^\top] = 0$, giving $HC + CH^\top = Q$. Conversely, if H is Hurwitz, the continuous Lyapunov equation has a unique $C \succeq 0$, and the OU process admits the stationary Gaussian with that covariance (standard OU theory).

[Weight–representation correspondence under local Boltzmann/OU regime] Let $a_\ell(\theta_\ell, x)$ be the activation vector at layer ℓ for input x, and fix a distribution over inputs with mean-zero feature $\bar{a}_\ell(\theta^{,x)=0}$ (or subtract the empirical mean). Assume within an epoch the parameter fluctuation $\Delta_\ell := \theta_\ell - \theta_\ell$ follows the OU approximation with stationary covariance

bioRxiv preprint doi: https://doi.org/10.64898/2026.01.30.702864; this version posted February 2, 2026. The copyright holder for this preprint (which was not certified by peer review) is the author/funder, who has granted bioRxiv a license to display the preprint in perpetuity. It is made available under aCC-BY 4.0 International license.

C_ℓ (Lemma 12). If a_ℓ is differentiable in θ_ℓ and we linearize in a neighborhood of θ_ℓ,

$$a_\ell(\theta_\ell, x) \;\approx\; a_\ell(\theta_\ell^{,x) + J_\ell(x)\,\Delta_\ell, \qquad J_\ell(x) := \frac{\partial a_\ell(\theta_\ell^{,x)}}{\partial \theta_\ell},}$$

then the representational covariance (over both parameter fluctuations and inputs) satisfies

$$\mathrm{Cov}[a_\ell] \;\approx\; \mathbb{E}_x\big[J_\ell(x)\,C_\ell\,J_\ell(x)^\top\big].$$

In particular, if $J_\ell(x)$ varies weakly with x (or we replace it by $J_\ell := \mathbb{E}_x[J_\ell(x)]$), then $\mathrm{Cov}[a_\ell] \approx J_\ell\,C_\ell\,J_\ell^\top$.

Let $\tilde{a}_\ell(\theta_\ell, x) := a_\ell(\theta_\ell, x) - \mathbb{E}_{x,\Delta_\ell}[a_\ell(\theta_\ell, x)]$. Under the linearization $\tilde{a}_\ell \approx J_\ell(x)\,\Delta_\ell +$ (zero-mean residual). Taking covariance over the joint law (independent x and Δ_ℓ),

$$\mathrm{Cov}[a_\ell] \;\approx\; \mathbb{E}_x\big[J_\ell(x)\,\mathbb{E}[\Delta_\ell\Delta_\ell^\top]\,J_\ell(x)^\top\big] \;=\; \mathbb{E}_x\big[J_\ell(x)\,C_\ell\,J_\ell(x)^\top\big].$$

If $J_\ell(x)$ is replaced by its mean J_ℓ (e.g., by linear response or a fixed probe set), the approximation reduces to $J_\ell C_\ell J_\ell^\top$.

Consequence (entropy–modularity link in representations). From Theorem 12 and the Gaussian OU covariance C_ℓ (Lemma 12), the activation covariance inherits block structure from C_ℓ through J_ℓ. Under fixed marginal variances, increasing within-block correlations (higher modularity) decreases $\det \mathrm{Cov}[a_\ell]$ by Hadamard's inequality, thus lowering differential entropy of a_ℓ. This matches the parameter-space result and provides the observable bridge.
